# A coral among stars: A new octocoral family (Anthozoa, Octocorallia, Malacalcyonacea) from seamounts in the tropical eastern Pacific

**DOI:** 10.3897/zookeys.1283.191899

**Published:** 2026-06-30

**Authors:** Odalisca Breedy, Catherine S. McFadden, Catalina Murillo-Cruz, Ana-Belén Yánez-Suárez, Katleen Robert, Andrea M. Quattrini

**Affiliations:** 1 Centro de Investigación en Ciencias del Mar y Limnología; Museo de Zoología, Centro de Investigación en Biodiversidad y Ecología Tropical, Universidad de Costa Rica, P. O. Box 11501-2060, San José, Costa Rica, Smithsonian Tropical Research Institute, Republic of Panama Department of Invertebrate Zoology, National Museum of Natural History, Smithsonian Institution Washington United States of America; 2 Centro de Investigación en Estructuras Microscópicas, Universidad de Costa Rica, San José, Costa Rica Department of Biology, Harvey Mudd College Claremont United States of America; 3 Department of Biology, Harvey Mudd College, Claremont, CA 91711-5990, USА Centro de Investigación en Ciencias del Mar y Limnología; Museo de Zoología, Centro de Investigación en Biodiversidad y Ecología Tropical, Universidad de Costa Rica San José Costa Rica; 4 Marine Institute, Memorial University of Newfoundland and Labrador, St John’s, Newfoundland, Canada Centro de Investigación en Estructuras Microscópicas, Universidad de Costa Rica San José Costa Rica; 5 Department of Invertebrate Zoology, National Museum of Natural History, Smithsonian Institution, Washington DC, USА Marine Institute, Memorial University of Newfoundland and Labrador St John’s Canada

**Keywords:** *28S* rDNA, Biodiversity, COI, deep-sea coral, integrative taxonomy, Isla del Coco, new family, new genus, phylogeny, UCEs

## Abstract

Existing studies highlight the remarkable diversity of life forms inhabiting deep-sea environments, despite the extreme conditions that characterize them. Here, a new family of gorgonian octocorals is described using an integrative taxonomic approach, combining morphological and genetic evidence. The organisms were obtained from seamounts off the insular shelf of Isla del Coco and three geological features of the Costa Rica Pacific margin, during two expeditions using the ROV *SuBastian* at depths of 360–529 m. Laurinqueidae fam. nov. is characterized by large irregular-flabellate colonies, with branching that is irregularly lateral, dense, and occasionally anastomosing. Thin coenenchyme includes a sclerome composed of rods and spindles with simple or complex tubercles; the axial sheath has rods and radiates. Polyps are tubular and conspicuously elongated, partially retractile into short cylindrical or dome-shaped mounds and arranged all around the branches. The anthocodiae have tuberculate rods and spindles arranged as points and an inconspicuous collaret. Colonies are bright yellow when alive. Phylogenetic analysis of two mitochondrial genes, *mtMutS* and *COI*, and the nuclear *28S rDNA* did not clarify the position of the taxon, with each single-gene marker placing it in a different family. Phylogenomic analysis of ultraconserved elements and exons strongly supports its placement in a unique clade, sister to Eunicellidae. The colony growth form and sclerome differ markedly from members of that family. Herein we describe *Laurinque elenya***gen. et sp. nov**. and place this unique lineage in the new family Laurinqueidae fam. nov. This study highlights the extreme incongruence among single-marker gene trees and the need for phylogenomic analyses based on hundreds of genes to elucidate the relationships among deeply divergent lineages of octocorals.

## Introduction

The deep ocean is defined as the marine region located below 200 m in depth and covers approximately 66% of the planet’s surface (Bell et al. 2025). Due to the logistical challenges involved in accessing these areas and the high costs associated with oceanographic expeditions, the available information remains limited and geographically biased toward certain regions (Amante and Eakins 2009; Bell et al. 2025). Nevertheless, existing studies highlight the remarkable diversity of life forms that inhabit these environments, despite the extreme environmental conditions that characterize them, such as high pressure, low temperatures, and limited to no light availability (Sánchez et al. 2024).

Octocorals are important components of the deep ocean ecosystems and constitute one of the most important foundation organisms (Buhl-Mortensen et al. 2010; Sánchez 2017). Dense aggregations of colonies form Marine Animal Forests (Orejas et al. 2022) and coral gardens (OSPAR 2022), generating complex, three-dimensional habitats that support numerous other taxa and have been identified as Vulnerable Marine Ecosystems (VMEs) (FAO 2019). Recent oceanographic campaigns (2019, 2023) exploring the seamounts off the insular shelf of Isla del Coco and the Pacific margin of Costa Rica revealed several undocumented taxa of deep-water octocorals (Cairns et al. 2026). The aim of the present study is to describe an octocoral found forming dense coral gardens on extensive aggregations of brittle stars (300–500 m in depth). We use an integrative taxonomic approach, combining morphological and molecular evidence to determine its phylogenetic placement and ecological relevance. Phylogenetic analyses of single-gene mitochondrial and nuclear markers (*mtMutS*, *COI*, *28S rDNA*) and phylogenomic analyses of ultraconserved elements and exons (collectively called UCEs) confirm the evolutionary uniqueness of this lineage, for which we describe a new species, genus and family.

## Materials and methods

### Study sites and collection methods

**Figure 1.**
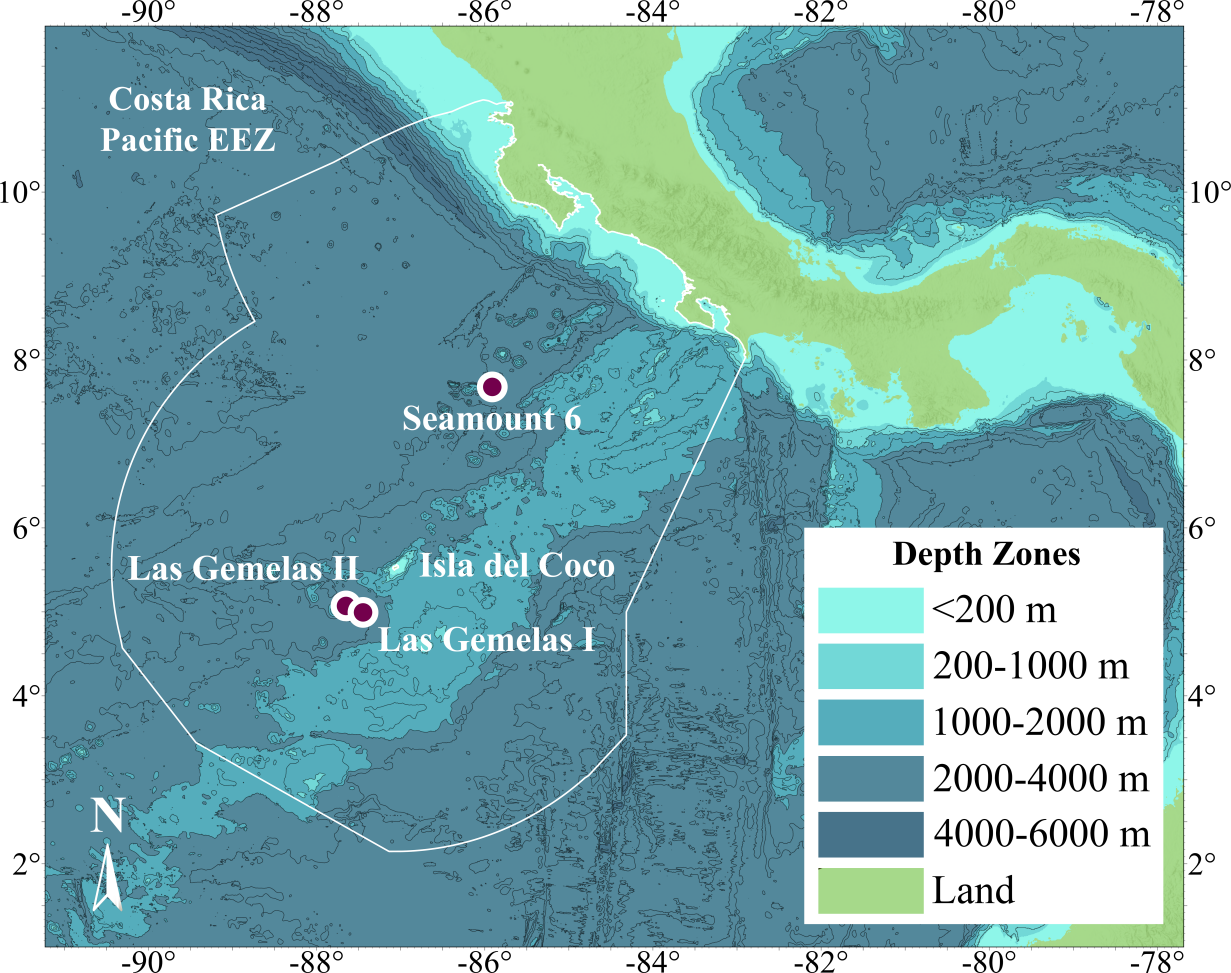
Map showing the geographic location of the collected samples within the Pacific Exclusive Economic Zone (EEZ) of Costa Rica. Bathymetric contours, drawn at 500 m intervals, and depth zones are based on the GEBCO_2022 Grid (GEBCO Compilation Group 2022). Made by Beatriz Naranjo (UCR).

The specimens were collected at four localities (Fig. 1 MAP) with the ROV *SuBastian* during two different expeditions: R/V *Falkor* (FK190106) to the Pacific margin of Costa Rica and R/V *Falkor (too)* (FKt 230918) to the Gemelas (I, II) seamounts, southwest of Cocos Island National Park, Costa Rica.

The colonies were photographed *in situ*, collected, and once retrieved they were kept alive for photographs and preliminary analyses. Small fragments were removed from the specimens and preserved in 95% ethanol for molecular studies; samples from Gemelas II were kept at –80 °C. The colonies were fixed in 95% ethanol or dried (in the case of large colonies) for morphological examination. The holotype and paratypes are deposited in the Museo de Zoología, Universidad de Costa Rica (**MZUCR**) del Centro de Investigación en Biodiversidad y Ecología Tropical, UCR.

### Morphological analysis

External characters of the colonies were analyzed from *in situ* photographs and dissection of collected samples under a stereoscope. Size of the *in situ* colonies was estimated by the paired laser scale (10 cm) of the ROV. For internal characters, sclerites from polyps and coenenchyme were obtained by dissolving the tissue in 5% sodium hypochlorite; dissociated sclerites were washed several times in distilled water until organic matter was completely removed, dehydrated with 100% ethanol, and subsequently dried. Arrangement of colony and polyp sclerites was determined by dissection and observation under a stereoscope. Sclerites were prepared for light microscopy, mounted in glycerin, and photographed with an Olympus LX 51 inverted microscope. For scanning electron microscopy (SEM), sclerites were mounted on aluminium stubs by double-stick carbon tape and silver paint, then sputter-coated with gold, 30–60 nm layer, in EMS 550X Ion Coater; the images were obtained using a FESEM Zeiss Sigma 300 (at 15 kV) in the Centro de Investigación en Estructuras Microscópicas, UCR (**CIEMIC**) and JEOL JSM-IT500 (at 15 kV) in the Centro de Investigación en Ciencia e Ingeniería de Materiales, UCR (**CICIMA**). Measurements of the sclerites were obtained from optical and SEM images. Taxonomic terminology follows Bayer et al. (1983).

### Molecular analysis

For the barcoding gene analysis, DNA was extracted from ethanol-preserved tissues with the DNeasy Blood and Tissue Kit (Qiagen, Germany) according to the manufacturer’s instructions and kept at –20 °C until further processing. A partial region of the mitochondrial mismatch repair gene (*mtMutS*) was amplified with ND42599F (5'-GCCATTATGGTTAACTATTAC- 3'; France and Hoover 2002) and MUT3458R (5'-TSGAGCAAAAGCCACTCC-3'; Sánchez et al. 2003), the mitochondrial cytochrome oxidase I gene (*COI*) with the primers COII8068F (5'-CCATAACAGGACTAGCAGCATC-3'; McFadden et al. 2004) and COIOCTR (5'-ATCATAGCATAGACCATACC-3'; France and Hoover 2002); and the *28S* nuclear ribosomal gene with 28S-Far (5'-CACGAGACCGATAGCGAACAAGTA-3') and 28S-Rar (5'-TCATTTCGACCCTAAGACCTC-3') (McFadden and van Ofwegen 2012) (Table 1).

**Table 1.** Molecular analysis of *Laurinque elenya* gen. et sp. nov. N/A: not available.

**MZUCR**	**Field number**	**Accession numbers**			
		***mtMutS***	***28S***	***COI***	**UCEs**
3884	FKt 230918-693	PZ137000	PZ102192	PZ101450	SAMN60142225
4022	FKt 230918-786	PZ137002	N/A	PZ101453	SAMN60142226
4080	FKt 230918-665	PZ137003	PZ102194	PZ101454	N/A
3088	FK190106 S0227-Q2	N/A	PZ102191	N/A	N/A
3892	FKt 230918-685	PZ137001	PZ102193	PZ101452	N/A
3886	FKt 230918-653	N/A	N/A	PZ101451	N/A

All the reactions were carried out in 25 μl volume with 15–45 ng DNA, 1.25 units Taq DNA polymerase (DreamTaq, Thermo Scientific, Waltham, MA), 1X DreamTaq Buffer, 0.2 mM of each dNTP, 0.2–0.3 μM of each primer, 0.1 mg/mL of BSA and 0.2–1.0 mM MgCl_2_ was added when necessary to optimize amplification. The amplification protocol for *mtMutS* consisted of 2 min of initial denaturation at 95 °C followed by 35 cycles of 30 sec at 95 °C, annealing at 50 °C for 30 sec, extension at 72 °C for 1 min and a final extension at 72 °C for 5 min, and for *COI* and *28S* was 5 min of initial denaturation at 95 °C followed by 35 cycles of 30 sec at 94 °C, annealing at 49 °C–50 °C for 45 sec, extension at 72 °C for 1 min and a final extension at 72 °C for 5 min. The resulting PCR products were purified and sequenced by Macrogen Inc. (Seoul, Korea), using the same forward and reverse PCR primers.

To obtain low-coverage genomic data for two specimens (MZUCR 3884, ID 693; MZUCR 4022, ID 786), DNA was extracted using a Qiagen DNeasy protocol and then quantified with a Qubit fluorometer and high-sensitivity assay reagents. DNA was then sheared to a target size range of 400–800 bp using sonication (Q800R QSonica). A total of 100 ng of DNA per sample was included in library preparation using a NEBNext Ultra II DNA Library Prep Kit. Library preparation followed the manufacturer’s protocol with some modifications: the reaction volume was reduced by half, 5 μl of iTru Y-yoke adaptor (Glenn et al. 2019) was used instead of NEBNext Adaptor, adaptor ligation time was 30 minutes, bead cleanups were performed with KAPA Pure Beads, iTru i5 and i7 indices (Glenn et al. 2019) were used, and 12 cycles of PCR enrichment were conducted. DNA concentration after library preparation was quantified with a Qubit dsDNA HS Assay Kit (ThermoFisher), and size distributions after library preparation were assessed on a TapeStation (Agilent Technologies). Molecular work was conducted in Smithsonian Institution’s Laboratories of Analytical Biology. Samples were sequenced on an Illumina NovaSeq X Plus (150 bp paired-end reads) (Table 1).

### Phylogenetic analyses

Sequences for *mtMutS* were aligned with the taxon-comprehensive dataset of McFadden et al. (2022) for order Malacalcyonacea; reference alignments for *28S rDNA* and *COI* were compiled to include representative sequences for as many of the same taxa as possible (Suppl. material 1). Sequences were aligned using MAFFT v. 5 (Katoh et al. 2005). Maximum likelihood trees were constructed for each gene separately using IQTree v. 2.3.1 (Minh et al. 2020) with the model of evolution identified by ModelFinder (Kalyaanamoorthy et al. 2017) (*mtMutS*: TVM+F+I+G4; *COI*: HKY+F+I+G4; *28S rDNA*: GTR+F+I+G4), 1000 ultrafast bootstrap replicates (Hoang et al. 2018) and estimation of the SH-like approximate likelihood ratio (SH-aLRT) (Guindon et al. 2010). Bayesian analyses were run with MrBayes v. 3.2.1 (Ronquist et al. 2012), specifying the most equivalent models of evolution (*mtMutS*: GTR+I+G; *COI*: HKY+I+G; *28S rDNA*: GTR+I+G). MrBayes was run for 5 × 10^6^, 7.5 × 10^6^ or 10 × 10^6^ generations for *COI*, *mtMutS* and *28S rDNA* respectively (until standard deviation of split frequences <0.01) with a burn-in of 25% and default Metropolis coupling parameters.

### UCE and exon analyses

We obtained UCEs from genomic data as in Quattrini et al. (2024). Briefly, demultiplexed reads were trimmed using fastp (Chen et al. 2018) and assembled using Spades v. 3.13.0 (Bankevich et al. 2012). Spades contigs were then passed to phyluce v. 1.7 (Faircloth 2016) to bioinformatically extract UCEs using the published bait set for octocorals (octo-v2, Erickson et al. 2021). The phyluce pipeline was used as described in the online tutorial (https://phyluce.readthedocs.io/en/latest/tutorials/tutorial-1.html) with some modifications following Quattrini et al. (2018, 2020). We combined previously published data from 393 octocoral samples and 12 outgroups (Quattrini et al. 2018, 2020, 2024; Untiedt et al. 2021; Erickson et al. 2021; McFadden et al. 2022; Quattrini et al. 2024) and each locus was aligned with MAFFT v7.130b (Katoh and Standley 2013) and internally trimmed with GBlocks (Castresana 2000). Phyluce was then used to create 50% and 75% taxon-occupancy matrices of aligned loci, which were then concatenated. Phylogenomic analyses were conducted using maximum likelihood in IQTree v. 2.1 (Minh et al. 2020) on the concatenated datasets with ultrafast bootstrapping (-bb 1000, Hoang et al. 2018). A partitioned model was used (-p). The best model of nucleotide substitution for each partition was found with ModelFinder (-m TESTMERGE, Kalyaanamoorthy et al. 2017) (Suppl. material 1). The 75% taxon-occupancy matrix was visualized in FigTree v 1.4.4 (Rambaut 2018). Both 50% and 75% treefiles and alignments are publicly available on FigShare (https://doi.org/10.25573/data.32246517).

## Results

### Systematic account

**Sub-Phylum Anthozoa Ehrenberg, 1834**

**Class Octocorallia Haeckel, 1866**

**Order Malacalcyonacea McFadden, van Ofwegen & Quattrini, 2022**

#### Family Laurinqueidae

Taxon classificationAnimaliaMalacalcyonaceaLaurinqueidae

Breedy, McFadden, Murillo-Cruz & Quattrini fam. nov.

1443D733-F0A2-57AB-8D8A-FAF7764D52C5

https://zoobank.org/F5209C95-9838-4D21-90CF-E2B5A9706DA7

##### Type genus.

*Laurinque* gen. nov.

##### Diagnosis.

Octocorals with a proteinaceous axis. Axis dark brown with a hollow, cross-chambered core surrounded by concentric layers of gorgonin; coenenchyme thin. Colonies erect, large, irregular-flabellate, reaching more than 1 m tall and 1.2 m wide. Branching throughout the colony irregularly lateral, dense, and occasionally anastomosing. Longitudinal furrows present along stem and branches. Polyps monomorphic, tubular and conspicuously elongated, especially at the base of the colony. Polyps partially retractile into the coenenchyme, producing short cylindrical or dome-shaped mounds arranged all around the branches and closely placed. Polyp sclerites are tuberculate rods and spindles arranged as points; collaret inconspicuous; tentacles and pharynx with rods. Sclerites of coenenchyme are rods and spindles with simple or complex tubercles; axial sheath with rods and radiates. Colonies bright yellow when alive, brownish when preserved or dry. Sclerites pale yellow. Azooxanthellate.

#### Genus Laurinque

Taxon classificationAnimaliaMalacalcyonaceaLaurinqueidae

Breedy, McFadden, Murillo-Cruz & Quattrini gen. nov.

4607C692-AEC7-595D-8D01-09B84A430D50

https://zoobank.org/A9BF739A-DEC6-455A-A962-E9F61A2256F3

##### Type species.

*Laurinque elenya* sp. nov. by monotypy.

##### Type locality.

Las Gemelas II, Isla del Coco, Costa Rica.

##### Generic diagnosis as for the family.

##### Etymology.

*Laurinquë*: neuter n. (Q) meaning golden tree in the Elvish language, Quenya (*The Lord of the Rings*, Tolkien 1955) in allusion to the bright yellow tree-like colonies. The golden octocoral gardens on fields of brittle stars evoke the mystic world of elves, where they spoke Quenya their ancient tongue (*The Lord of the Rings*, Tolkien 1955; *The Silmarillion*, Tolkien 1977).

#### Laurinque elenya

Taxon classificationAnimaliaMalacalcyonaceaLaurinqueidae

Breedy, McFadden, Murillo-Cruz, Yánez-Suárez, Robert & Quattrini sp. nov.

B977A774-E3C5-54CD-8D24-77D0E33B2899

https://zoobank.org/6E0BCF80-06A2-42E6-9EA7-1ACA7B7DF170

Figs 2, 3, 4, 5, 6

##### Type locality.

Las Gemelas II, Isla del Coco, Costa Rica.

##### Material examined.

***Holotype***. Costa Rica • one specimen, ethanol/dry preserved; off Isla del Coco, Las Gemelas II; 5.071715, -87.65116; 362.53 m depth; 16 Oct. 2023; R/V *Falkor (too)*, ROV *SuBastian*, S0601; MZUCR 3884, FKt 230918-693. ***Paratypes***. Costa Rica • (one specimen, ethanol preserved); off Isla del Coco, Las Gemelas II; 5.071503, -87.65332; 412.18 m depth; 16 Oct. 2023; R/V *Falkor (too)*, ROV *SuBastian*, S0601; MZUCR 4022, FKt 230918–786 • (one specimen, ethanol preserved); off Isla del Coco, Las Gemelas I; 4.994402, -87.448105; 404.77 m in depth; 15 Oct. 2023; R/V *Falkor (too)*, ROV *SuBastian*, S0600; MZUCR 4080, FKt 230918–665 • (one specimen, ethanol preserved); off Isla del Coco, Las Gemelas I; 4.992902, -87.446944; 359.87 m depth; 15 Oct. 2023; R/V *Falkor (too)*, ROV *SuBastian*, S0600; MZUCR 3892, FKt 230918–685 • (one specimen, ethanol preserved); off Isla del Coco, Las Gemelas I; 4.994389, -87.448117; 404.77 m depth; 22 Jan. 2019; R/V *Falkor (too)*, ROV *SuBastian*, S0600; MZUCR 3886, FKt 230918–653 • (one specimen, ethanol preserved); Eastern Pacific Ocean, Seamount 6; 7.679967, -85.911561; 529 m depth; R/V *Falkor*, ROV *SuBastian*, S0227; MZUCR 3088, FK190106 S0227-Q2.

##### Description.

**Figure 2.**
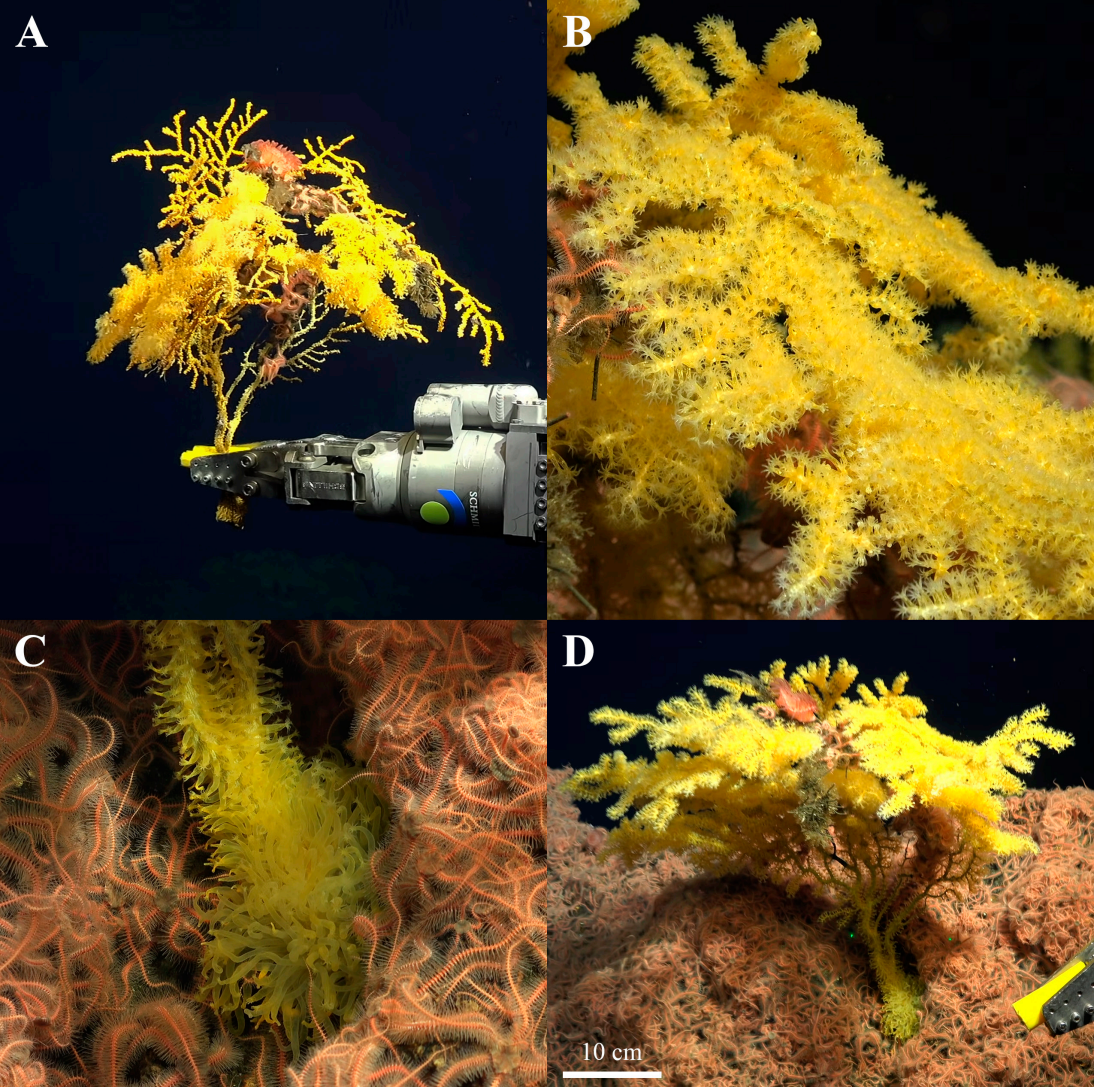
*Laurinque elenya* gen. et sp. nov., morphology of the holotype, *in situ*. **A**. Colony collection; **B**. Detail of polyps; **C**. Elongated polyps at the base of the colony; **D**. Complete colony, laser points separated 10 cm. Photographs by ROV *SuBastian* (FKt 230918).

***Colony***. The holotype is a large irregular-flabellate colony, up to 50 cm tall and 60 cm wide (Fig. 2A, D); the specimen was preserved dry, and a fragment was preserved in ethanol. Branching throughout the colony is irregularly lateral, dense, approximately in one plane, and occasionally anastomosing (Fig. 2A, B, D). The stem is ~3 cm long and 1.5 cm in diameter, and slightly flattened (Fig. 2A, C, D). It arises from an extended holdfast and produces several main branches, 0.6–1.0 cm in diameter. The branches bifurcate 20–30 times, producing branchlets 0.5–6.0 cm apart and 0.1–0.6 cm in diameter (Fig. 2A, B, D). Free ends are up to 6 cm long, 0.10–0.15 mm in diameter. The axis is proteinaceous, dark brown with concentric layers of gorgonin and a hollow cross-chambered core. The stem and holdfast are covered by elongated tubular polyps (Fig. 2C); the holdfast extends on the substrate under brittle stars. The holdfast of the collected colony is ~5 cm in diameter. The coenenchyme is thin, with parallel furrows on the main axis and branches which extend all along the colony (Fig. 6C); furrows are more evident on the dry fragment of the holotype. When the colony is preserved, dry or in ethanol, the coenenchyme easily detaches from the axis.

**Figure 3.**
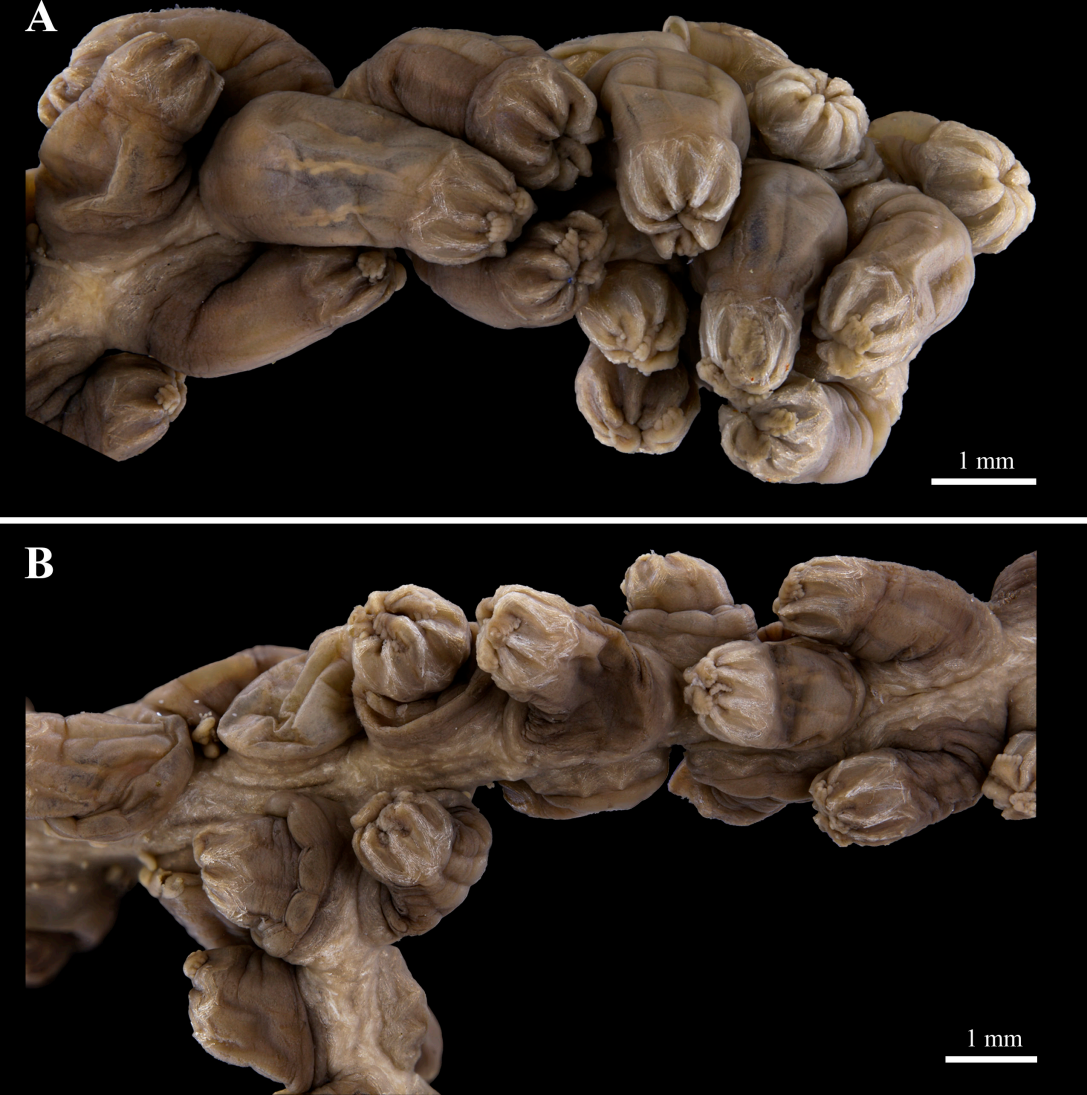
*Laurinque elenya* gen. et sp. nov. **A, B**. Holotype (ethanol preserved fragment) detail of anthocodiae and polyp arrangement. Photographs by Fiorella Vásquez (UCR).

**Figure 4.**
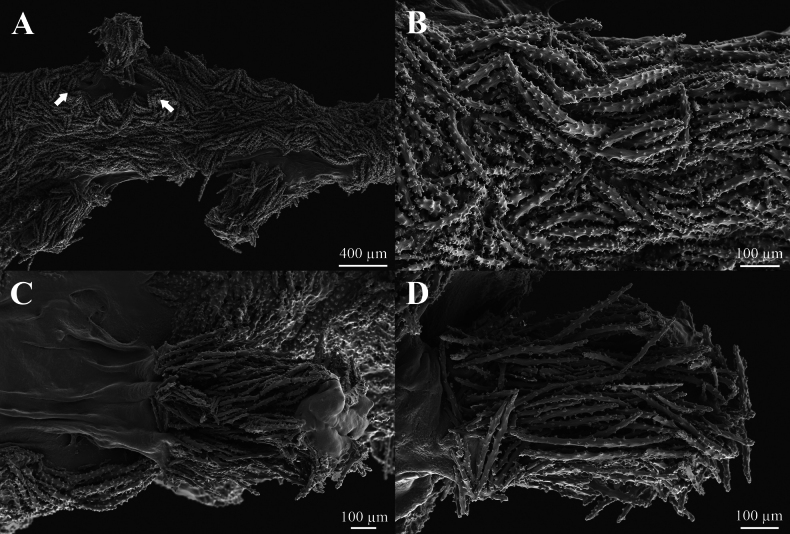
*Laurinque elenya* gen. et sp. nov., holotype, scanning electron micrographs from partially digested fragments. **A**. Branch showing coenenchymal sclerites and polyp mounds; **B**. Magnified image of the external coenenchyme; **C**. Anthocodia showing the nude neck zone; **D**. Anthocodial sclerites.

***Polyps***. The polyps are monomorphic, long, tubular, the neck zone without sclerites (Figs 3A, 3B, 4A, 4C); they can reach up to 5 mm long (Fig. 2B, C). The colony appears fuzzy due to the densely packed, expanded polyps, especially observed at the lower part of the stem and holdfast (Fig. 2A–D). Polyps are arranged in longitudinal rows on the stem and thicker branches, and all around the thin branches and branchlets. They are closely set and partially retract into short cylindrical or dome-shaped mounds, 0.5–1.0 mm tall and 1.0–1.5 mm in diameter (Fig. 3A, B). Polyps at the ends of branches are often in clusters of four or five polyps (Figs 2B, 3A, 3B). When alive, the long neck zone of the anthocodia is translucent, while the upper part and tentacles are of a bright yellow color (Figs 2B, 2C, 3A, 3B, 6A). When preserved in ethanol or dry, the color vanishes (Fig. 3A, B).

**Figure 5.**
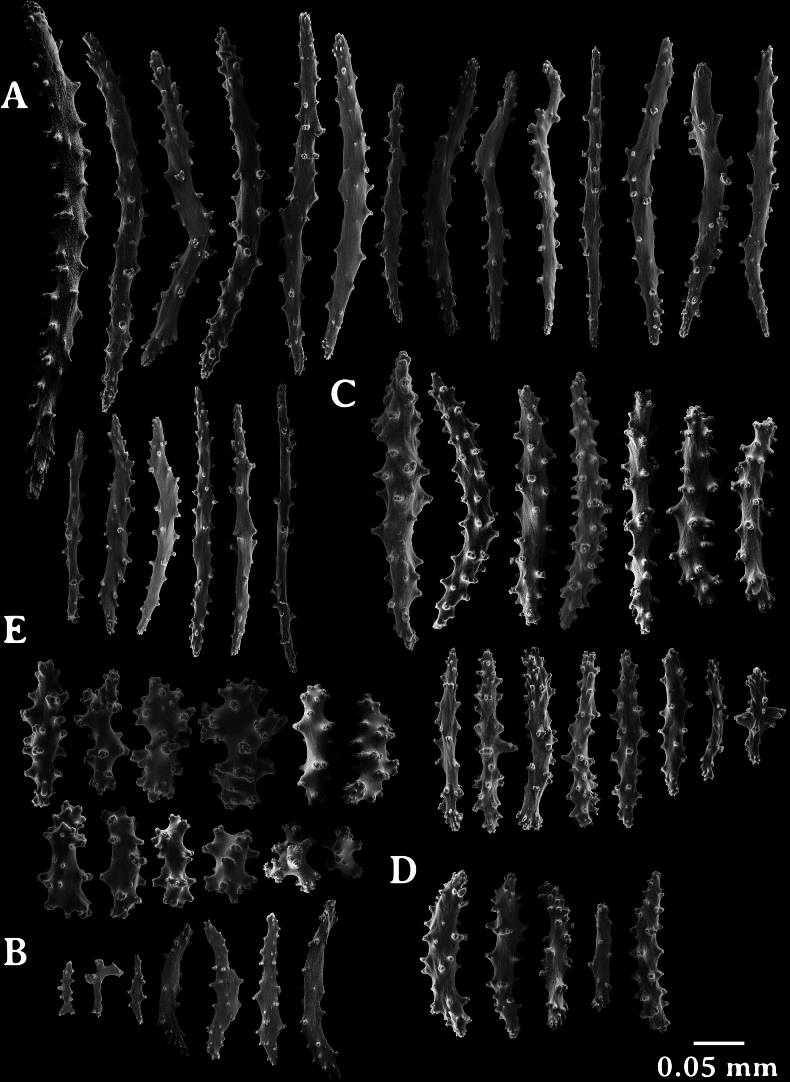
*Laurinque elenya* gen. et sp. nov., holotype, SEM micrographs. **A**. Anthocodial spindles; **B**. Polyp rods; **C, D**. External coenenchyme and polyp mound spindles; **E**. Internal coenenchyme radiates and short spindles.

***Sclerites***. Polyp sclerites are arranged as collaret and points (Fig. 4A, C, D). Points of long curved, straight spindles with short spines, and irregular spindles with short tubercles, 0.2–0.5 mm long and 0.02–0.04 mm wide (Figs 4C, 4D, 5A), arranged *en chevron* (Figs 3A, 3B, 4A, 4C, 4D); collaret is inconspicuous, composed of a few shorter, irregular spindles and rods found at the base of the points (Figs 3A, 3B, 4A, 4C, 4D, 5A, 5B). Tentacular and pharyngeal sclerites are flattened rods, slightly curved, with small warts and lobed or serrated irregular borders, 0.05–0.30 mm long and 0.02–0.03 mm wide (Fig. 5B). Coenenchyme includes various types of spindles (Fig. 4B): irregular, warty, tuberculate, 0.1–0.3 mm long and 0.03–0.08 mm wide (Fig. 5C), and shorter, straight or slightly curved, irregular warty spindles, 0.10–0.25 mm long and 0.02–0.035 mm wide (Fig. 5D). The same type of coenenchymal sclerites are arranged en chevron at the opercular rim of the polyp mounds (Fig. 4A). The axial sheath includes radiates and crosses, 0.06–0.10 mm and 0.04–0.06 mm, respectively (Fig. 5E). The coenenchyme is very thin, making the difference between the sclerites of the axial sheath and external coenenchyme difficult to determine.

**Figure 6.**
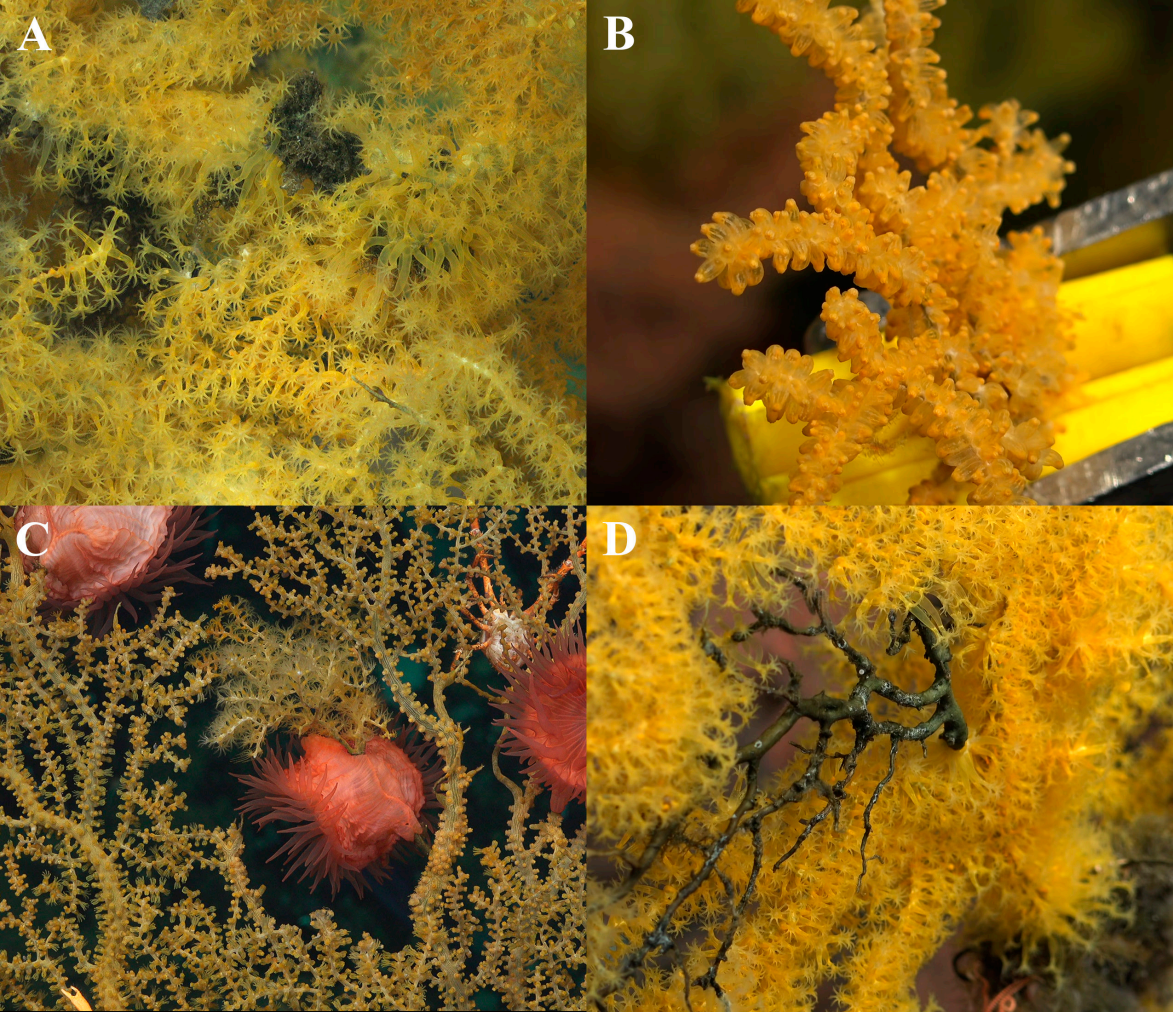
*Laurinque elenya* gen. et sp. nov., *in situ* colonies in Las Gemelas II. **A**. Detail of expanded polyps; **B**. Paratype MZUCR 4022 during collection showing retracted tentacles; **C**. Detail of branches, showing longitudinal furrows and retracted polyps; **D**. Expanded polyps and nude axes. Photographs by ROV *SuBastian*.

***Color***. The colony is bright yellow when alive (Fig. 2A–D), and of darker hue when preserved (Fig. 3A, B). The dry fragment of the holotype is dark brown. Sclerites are pale yellow. The preserved specimen dyed the ethanol brownish.

**Figure 7.**
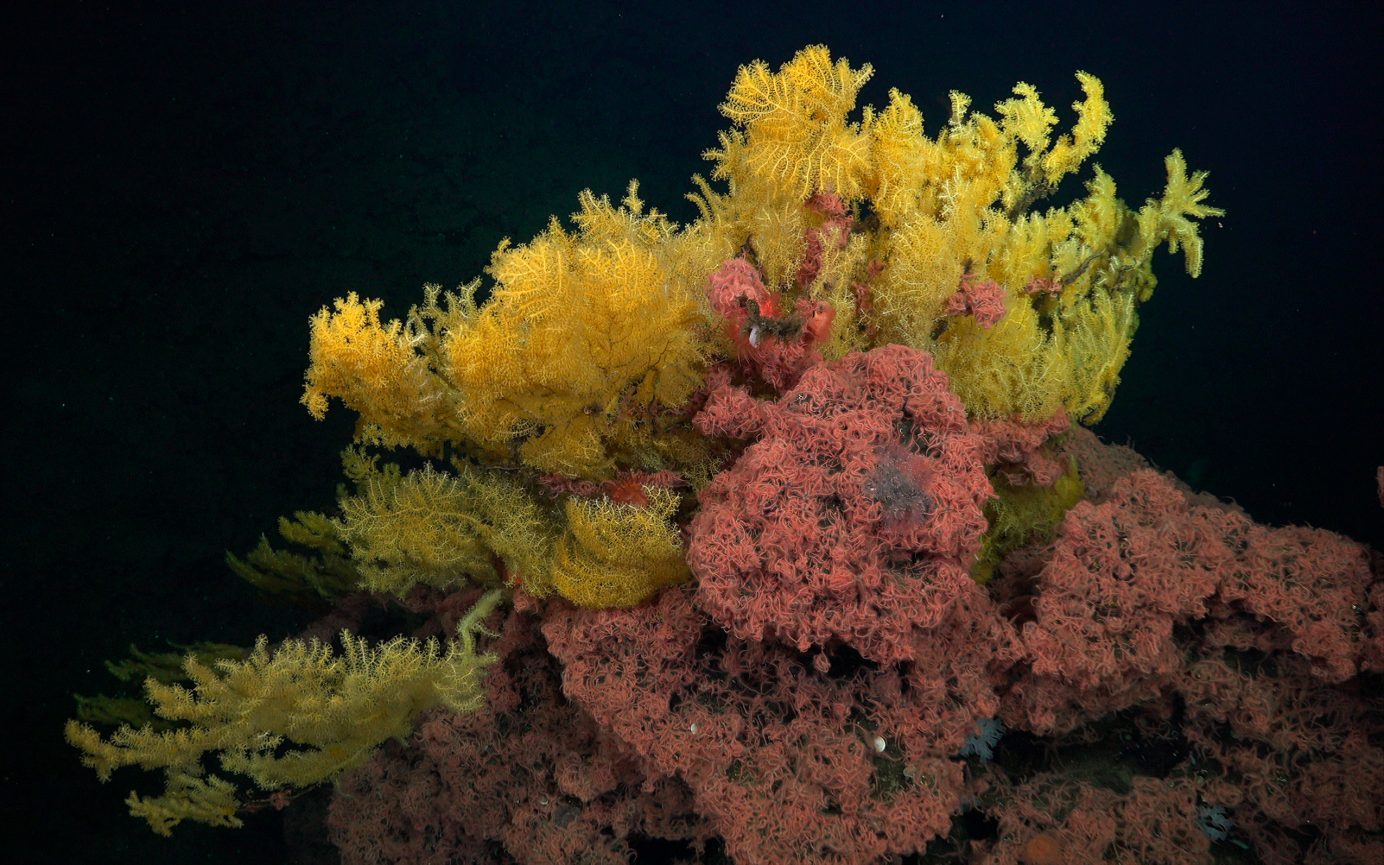
*In situ* colonies of *Laurinque elenya* gen. et sp. nov. in Las Gemelas II, showing the octocoral garden within the brittle stars mat (Ophiacanthidae, *Ophiacantha* sp.). Paratype MZUCR 4022 collected from one of these colonies. Photographs by ROV *SuBastian*.

***Variations***. The paratypes consist of a small colony with a single branch, 4 cm long (MZUCR 3892) attached to a rock; a complete colony 25 cm long, 11 cm wide (MZUCR 3088); and small fragments (MZUCR 4080, 4022) from larger colonies (Fig. 7B; paratype MZUCR 4022). All the paratypes are consistent in sclerite types with some variation in size, and colony morphology. The colonies observed at Las Gemelas I, II were larger and more frondose than the ones from Seamount 6. Eggs were found in the specimens collected in Las Gemelas II.

##### Habitat.

Large colonies of *L. elenya* gen. et sp. nov. were found forming extensive gardens on rocky substrate densely inhabited by brittle stars, *Ophiacantha* sp. in the family Ophiacanthidae, which completely covered the surface in Las Gemelas II (Fig. 7). The stems and holdfasts of the colonies were mostly buried beneath the mat of brittle stars (Figs 2C, 7). A similar substrate was documented in Las Gemelas I, although the colonies were more sparsely distributed along the sampled area. The depth range of the species lies within the extremely low oxygen zone (0.5–3.0 μmol kg^-1^ or 0.01–0.067 mL L^-1^), with water temperatures of 8.12–10.8 °C and salinity of 34.6–34.7 ppt. The species was also found scattered across Seamount 6, but we did not find gardens in this location.

##### Etymology.

*Elenya*: neuter adj. (Q) stellar, constellation, in allusion to the field of stars where the species was found.

##### Distribution.

Seamounts in the Eastern Tropical Pacific of Costa Rica (Fig. 1). The species was found at 362–412 m in depth at Las Gemelas I, II, and 529 m at Seamount 6, representing the deepest record so far.

##### Remarks.

*Laurinque elenya* gen. et sp. nov. was also observed in Panamá and the Galápagos Islands (OB, pers. obs.), but this occurrence needs to be corroborated.

## Molecular results

### Phylogenetic analysis of barcoding genes

**Figure 8.**
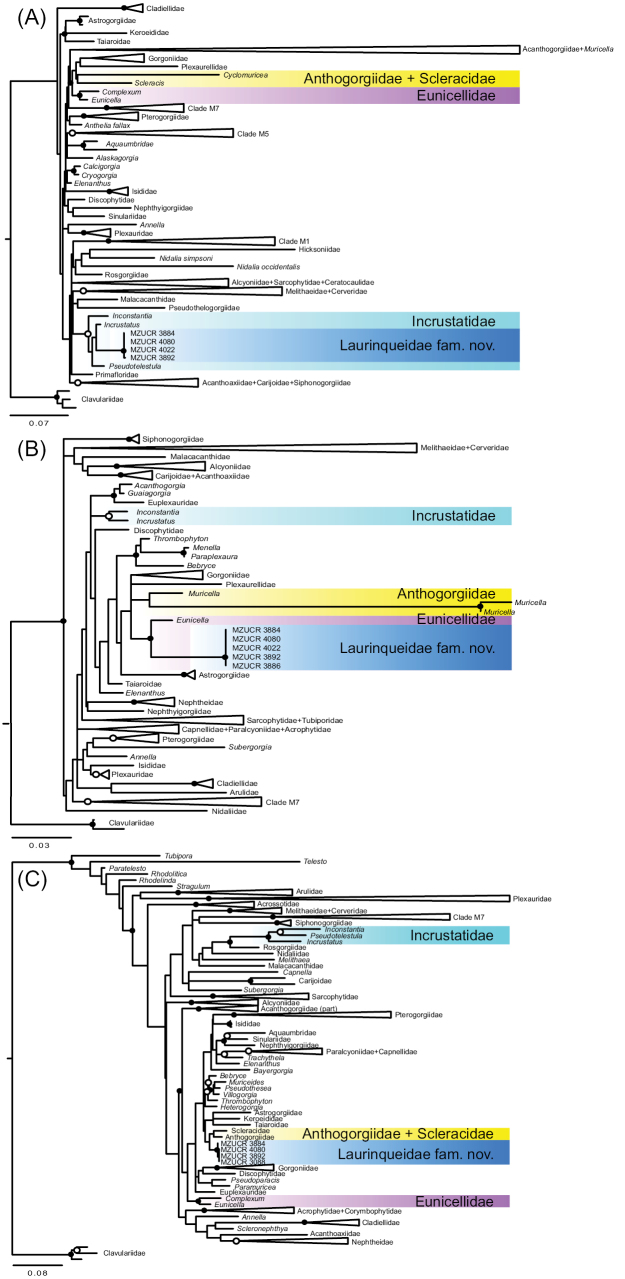
Maximum likelihood reconstructions of octocoral order Malacalcyonacea based on **A**. *mtMutS* (783 bp); **B**. *COI* (536 bp); **C**. *28S rDNA* (867 bp). Families recovered as sister to Laurinqueidae fam. nov. in different gene trees highlighted in color. Clades are collapsed to enhance readability; families and clades identified as in McFadden et al. 2022. All taxa included in trees are listed in Suppl. material 1. Circles at nodes: solid = ML UFboot p > 0.9, Bayesian pp > 0.9; open = ML UFboot p < 0.9, Bayesian pp > 0.9.

Phylogenetic reconstructions of the three different barcoding genes were highly incongruent, with each gene tree suggesting a different phylogenetic placement of *Laurinque elenya* gen. et sp. nov. (Fig. 8, Table 1). Results of maximum likelihood (ML) and Bayesian (MB) analyses for each gene were, however, in general agreement. The *mtMutS* gene tree placed *L. elenya* in family Incrustatidae McFadden, van Ofwegen & Quattrini, 2024 with strong support (ML: UFboot = 100%, SH-aLRT = 100; MB: pp = 0.83) (Fig. 8A). Within that family, both analyses suggested it was sister to the genus *Incrustatus* van Ofwegen, Häussermann & Försterra, 2007 (ML: UFboot = 85, SH-aLRT = 99; pp = 0.81). In sharp contrast, *COI* gene trees placed *L. elenya* in family Eunicellidae McFadden, van Ofwegen & Quattrini, 2024 with strong support from both approaches (ML: UFboot = 91%, SH-aLRT = 91; MB: pp = 1.0) (Fig. 8B). Gene trees for *28S rDNA* were poorly resolved (Fig. 8C). The ML analysis placed *L. elenya* sister to a clade of families Anthogorgiidae McFadden, van Ofwegen & Quattrini, 2024 plus Scleracidae McFadden, van Ofwegen & Quattrini, 2024 with only weak support (UFboot = 83%, SH-aLRT = 87) while the Bayesian analysis placed it among a poorly supported (pp = 0.61) clade of gorgonians (former Holaxonia) that included both Eunicellidae and [Anthogorgiidae + Scleracidae] but with no support for a sister relationship between *L. elenya* and either of those clades.

### Phylogenomic analysis of UCEs

**Figure 9.**
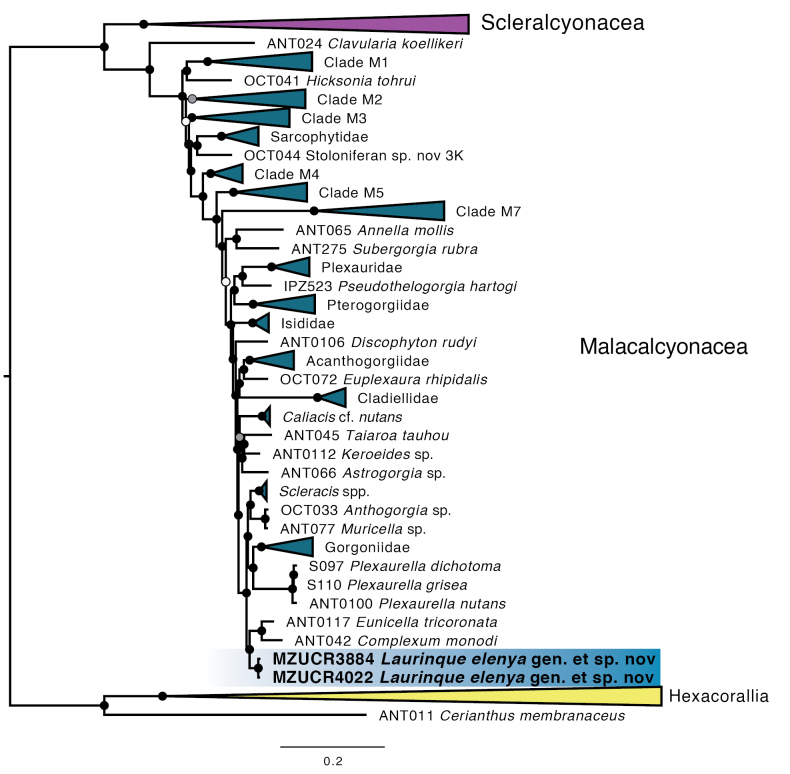
Maximum likelihood phylogeny of octocorals based on UCEs (75% matrix, 153 loci, 37,654 bp). Outgroups are included. Node support values, represented by circles, include ultrafast bootstraps > 95% (black), 85-95% (grey) and <85% (white).

We obtained 10,970,942 (ID 786, MZUCR 4022) and 27,773,876 (ID 693, MZUCR 3884) (Table 1) paired-end Illumina reads for each specimen. A total of 1934-2236 UCEs (727-2392 median length) UCEs were obtained. The phylogenomic analysis based on 153 loci (75% taxon occupancy, 37,654 bp) strongly supported *Laurinque elenya* gen. et sp. nov. as the sister group to family Eunicellidae (Fig. 9), in agreement with the *COI* gene tree (Fig. 8B). The 50% taxon-occupancy (1,385 loci, 263,175 bp) tree also supports this phylogenetic relationship.

## Discussion

The combination of morphological characteristics—growth form, polyp shape and arrangement, and sclerome—supports the establishment of a new family for these octocorals. When alive, the colonies could be confused with species of the genus *Acanthogorgia* Gray, 1857 because of the color, delicate colony appearance and thin coenenchyme, but the coenenchymal sclerites differ and the characteristic long intertentacular spines of that genus are absent in the new family. In addition, the polyps of *Acanthogorgia* are heavily armed with sclerites that prevent the polyp from retracting, unlike the sclerite-free neck zone of the polyps of *Laurinque* gen. nov. that allows retraction. *Laurinque* gen. nov. could also be confused with species of Anthogorgiidae, another family characterized by heavily armed, non-retractile polyps.

From the molecular point of view, the species is related to the family Eunicellidae McFadden, van Ofwegen & Quattrini, 2024, however, the sclerites are different and the balloon-club sclerites characteristic of the genus *Eunicella* Verrill, 1869 are not present. The genus *Complexum* van Ofwegen, Aurelle & Sartoretto, 2014 that is also included in Eunicellidae lacks an axis and does not share any morphological characteristics with Laurinqueidae fam. nov..

The phylogenetic reconstructions based on three genetic markers typically used for DNA barcoding of octocorals (McFadden et al. 2014) were unable to resolve the relationship of *Laurinque* gen. nov. to other families of octocorals. Moreover, the three different gene trees were highly incongruent with one another, each suggesting a different familial placement (Fig. 8). Most surprising was the strong incongruence between the two mitochondrial gene markers, *mtMutS* and *COI.* While the gene tree for *COI* recovered the same topology as the UCE tree, supporting *Laurinque* gen. nov. as the sister to Eunicellidae, *mtMutS* strongly supported the placement of *Laurinque* gen. nov. in Incrustatidae, a family of stoloniferous and encrusting soft corals that lack axes. Previous studies have noted the frequent incongruence between mitochondrial and nuclear gene phylogenetic reconstructions at all taxonomic levels in octocorals, suggesting that mitochondrial trees may reflect the evolutionary history of the organelle rather than the species (Quattrini et al. 2023; Morrissey et al. 2025). In addition, selection appears to act strongly on mitochondrial genomes (Ramos et al. 2023) and different lineages of octocorals exhibit very different rates of mitochondrial gene evolution (Muthye et al. 2022). While the mitochondrial *mtMutS* gene typically resolves genus-level relationships well (McFadden et al. 2022), our results caution against its use for inferring family-level relationships among octocorals. Use of this gene alone to infer the phylogenetic placement of *Laurinque* gen. nov. would have resulted in a spurious assignment to a family with which it shares few if any diagnostic morphological characters.

## Supplementary Material

XML Treatment for Laurinqueidae

XML Treatment for Laurinque

XML Treatment for Laurinque elenya

## Additional information

### Artificial Intelligence (AI) use

The authors accept full responsibility for the content of the manuscript, including the disclosure of any use of AI.

No AI tools were used in the preparation of this manuscript.

### Author ORCIDs

O. Breedy https://orcid.org/0000-0001-5686-4164

C.S. McFadden https://orcid.org/0000-0002-8519-9762

C. Murillo-Cruz https://orcid.org/0000-0003-0218-7852

A.-B. Yánez-Suárez https://orcid.org/0009-0000-4415-0447

K. Robert https://orcid.org/0000-0003-0119-5359

A.M. Quattrini https://orcid.org/0000-0002-4247-3055

### Data availability

All of the data that support this study are available in the main text, Suppl. material 1 material and FigShare: https://doi.org/10.25573/data.32246517 and on NCBI Bioproject: PRJNA1466563.

## Supplementary materials

Supplementary material 1 Sequences included in phylogenetic analysis of Malacalcyonacea Data type xlsx https://binary.pensoft.net/file/1702429This dataset is made available under the Open Database License (http://opendatacommons.org/licenses/odbl/1.0/). The Open Database License (ODbL) is a license agreement intended to allow users to freely share, modify, and use this Dataset while maintaining this same freedom for others, provided that the original source and author(s) are credited. Odalisca Breedy, Catherine S. McFadden, Catalina Murillo-Cruz, Ana-Belén Yánez-Suárez, Katleen Robert, Andrea M. Quattrini
